# Supplementary material for: Structure and genome editing of type I-B CRISPR-Cas
Source: Nat Commun. 2024 May 15;15:4126. doi: 10.1038/s41467-024-48598-2 (PMC11096372; doi:10.1038/s41467-024-48598-2)
Supplement: Supplementary file 1 — Supplementary information [file 41467_2024_48598_MOESM1_ESM.pdf]

Supplementary Information

Structure and genome editing of type I-B CRISPR-Cas

Meiling Lu[1, 2, 9]*, Chenlin Yu[1, 9], Yuwen Zhang[1], Wenjun, Ju[1], Zhi Ye[1], Chenyang, Hua[1], Jinze Mao[3], Chunyi Hu[4, 5], Zhenhuang Yang[6]*, Yibei Xiao[2,7,8]*

[1] Department of Biochemistry, School of Life science and technology, China Pharmaceutical University, Nanjing 211198, China

[2] State Key Laboratory of Natural Medicines, China Pharmaceutical University, Nanjing 211198, China,

[3] Nanjing Foreign Language School, Nanjing 210008, China

[4] Department of Biological Sciences, Faculty of Science, National University of Singapore, Singapore 117543, Singapore

[5] Precision Medicine Translational Research Programme (TRP), Department of Biochemistry, Yong Loo Lin School of Medicine, National University of Singapore, Singapore 117543, Singapore

[6] Institute for Hepatology, National Clinical Research Center for Infectious Disease, Shenzhen Third People's Hospital, Shenzhen, Guangdong 518112, China

[7] Department of Pharmacology, School of Pharmacy, China Pharmaceutical University, Nanjing, 211198, China

[8] Chongqing Innovation Institute of China Pharmaceutical University, Chongqing 401135, China

[9] These authors contributed equally: Meiling Lu, Chenlin Yu.

* To whom correspondence should be addressed. Email : lumeiling@cpu.edu.cn ; yanginchina@hotmail.com ; yibei.xiao@cpu.edu.cn.

Supplementary Table 1-5
Supplementary Figure 1-9

Supplementary Table 1. Synthetic DNA Sequences

Name	Sequence (5' to 3')	Note
PF-885	CGGGATCCATGCCGAAAACCCAAGCGG	SynCas8-Cas7-Cas5 PCR amplification
PR-885	CCGCTCGAGTTAGCCCGGCAGTTG	SynCas8-Cas7-Cas5 PCR amplification
PF-6	CGGGATCCATGAACTTCATCGACC	SynCas6 PCR amplification
PF-6	CCCAAGCTTTTAGCCCAGGGTC	SynCas6 PCR amplification
PF-11	GGAATTCCATATGATGACCGTGGCGACCAC	SynCas11 PCR amplification
PR-11	CCGCTCGAGTTATTCGGTCTCGCCTTG	SynCas11 PCR amplification
PF-Mix-PAM	GATCCNNNTTTATCACCGTGTCCCCAATCTGGATATTTTGTGTC	NNN-PAM-protospacer
PR-Mix-PAM	TCGCGACACAAAATATCCAGATTGGGGACACGGTGATAAANNNG	NNN-PAM
PF-6-FAM	TAATACGACTCACTATAGG	5'-6-FAM labelled forward primer
PR-CY5	GCTAGTTATTGCTCAGCGG	3'-CY5 labelled reverse primer
PF-97	TAATACGACTCACTATAGGTCGCGGATCC	59 bp ANN dsDNA amplification
PR-97	GCTAGTTATTGCTCAGCGGTGGTGCTCGAGACAC	59 bp ANN dsDNA amplification
PF-161	TAATACGACTCACTATAGGCGCGGCAGCCATATGGCTAGCATG	PAM library plasmids amplification
PR-161	GCTAGTTATTGCTCAGCGGGATCTCAGTGGTGGTGGTGGTGGTGC	PAM library plasmids amplification
PF-TRAC	TTCCACCTCAAAACATTGGTTCCGTC	For DNA lesion analysis
PR-TRAC	CCCTCTGACCTCTGCCCTAACTGG	For DNA lesion analysis
PF-G102A	CCCGGTTGCTAACAGCGGCAAGGAGTTTGCGA	For the expression plasmid construction of the of Cas5 G102A mutant
PR-G102A	CTGTTAGCAACCGGGTAGTTGTGCAGTTGC	For the expression plasmid construction of the of Cas5 G102A mutant
PF-N103A	GTTGGTGCCAGCGGCAAGGAGTTTGCG	For the expression plasmid construction

		of the of Cas5 N103A mutant
PR-N103A	TTGCCGCTGGCACCAACCGGGTAGTTGTGCA<br>G	For the expression plasmid construction of the of Cas5 N103A mutant
CRISPR array	GTGTCCAAACCATTGATGCCGTAAGGCGTTG<br>AGCACTTTATCACCGTGTCCCCAATCTGGATA<br>TTTTGTGTGTGTCCAAACCATTGATGCCGTAA<br>GGCGTTGAGCACTTTATCACCGTGTCCCCAA<br>TCTGGATATTTTGTGTGTGTCCAAACCATTGA<br>TGCCGTAAGGCGTTGAGCAC	*Syn*repeat-spacer-repeat-spacer-repeat
59bp Target DNA strand	TCGCGGATCCATGTTTATCACCGTGTCCCCAA<br>TCTGGATATTTTGTGTCTCGAGCACCA (TS)<br>AGCGCCTAGGTACAAATAGTGGCACAGGGGT<br>TAGACCTATAAAACACAGAGCTCGTGGT<br>(NTS)	ATG PAM-protospacer
Cas8b	ATGGGCAGCAGCCATCACCATCATCACCACC<br>ACCACAGCCAGTGGAGCCATCCGCAGTTTGA<br>AAAAGGTGGTGGTAGCGGTGGTGGTTCAGG<br>TGGTAGTGCATGGTCACACCCTCAGTTTGAG<br>AAACTGGAAGTGCTGTTCCAGGGTCCGGGAT<br>CCATGCCGAAAACCCAAGCGGAGATCCTGAC<br>CCTGGACTTCAACCTGGCGGAACTGCCGAGC<br>GCGCAACACCGTGCGGGTCTGGCGGGTCTG<br>ATCCTGATGATTCGTGAGCTGAAGAAATGGC<br>CGTGGTTTAAGATCCGTCAAAAGGAGAAAG<br>ACGTGCTGCTGAGCATTGAAAACCTGGATCA<br>GTACGGTGCGAGCATCCAACTGAACCTGGAA<br>GGCCTGATTGCGCTGTTCGATCTGGCGTATCT<br>GAGCTTTACCGAGGAGCGTAAGAGCAAAAG<br>CAAGATCAAAGACTTCAAACGTGTTGATGAG<br>ATCGAAATTGAGGAAAACGGCAAGAACAAG<br>ATCCAGAAGTACTACTTCTACGACGTGATTAC<br>CCCGCAAGGTGGCTTTCTGGCGGGTTGGGAC<br>AAAAGCGATGGCCAGATCTGGCTGCGTATTT<br>GGCGTGATATGTTCTGGAGCATCATTAAGGGC<br>GTTCCGGCGACCCGTAACCCGTTTAACAACC<br>GTTGCGGTCTGAACCTGAACGCGGGCGACA<br>GCTTCAGCAAGGATGTTGAGAGCGTGTGGA<br>AAAGCCTGCAGAACGCGGAAAAGACCACCG<br>GTCAAAGCGGCGCGTTTTACCTGGGTGCGAT<br>GGCGGTTAACGCGGAAAACGTGAGCACCGA<br>CGATCTGATCAAATGGCAGTTCCTGCTGCAC	His_6 tag-Twin strep tag-PreScission site-Cas8

	TTCTGGGCGTTTGTTGCGCAAGTGTACTGCC<br>CGTATATTCTGGACAAGGATGGTAAACGTAA<br>CTTTAACGGCTATGTGATCGTTATTCCGGACA<br>TCGCGAACCTGGAGGACTTCTGCGATATTCT<br>GCCGGATGTGCTGAGCAACCGTAACAGCAA<br>AGCGTTCGGTTTTCGTCCGCAGGAAAGCGTT<br>ATCGACGTGCCGGAGCAAGGCGCGCTGGAA<br>CTGCTGAACCTGATCAAGCAGCGTATTGCGA<br>AGAAAGCGGGTAGCGGCCTGCTGAGCGATC<br>TGATCGTGGGTGTTGAGGTGATCCACGCGGA<br>AAAGCAGGGCAACAGCATCAAACTGCACAG<br>CGTTAGCTACCTGCAACCGAACGAGGAAAG<br>CGTGGACGATTATAACGCGATTAAGAACAGC<br>TACTATTGCCCGTGGTTCCGTCGTCAGCTGCT<br>GCTGAACCTGGTTAACCCGAAATTTGACCTG<br>GCGAGCCAAAGCTGGCTGAAGCGTCACCCG<br>TGGTACGGTTTTGGCGATCTGCTGAGCCGTAT<br>CCCGCAGCGTTGGCTGAAAGAGAACAACAG<br>CTATTTCAGCCACGACGCGCGTCAGCTGTTC<br>ACCCAAAAGGGTGACTTTGATATGACCGTGG<br>CGACCACCAAAACCCGTGAGTACGCGGAAA<br>TCGTTTATAAGATTGCGCAGGGTTTCGTGCTG<br>AGCAAGCTGAGCAGCAAACACGACCTGCAA<br>TGGAGCAAGTGCAAAGGCAACCCGAAACTG<br>GAGCGTGAATACAACGATAAGAAAGAGAAG<br>GTGGTTAACGAAGCGTTTCTGGCGATCCGTA<br>GCCGTACCGAAAAACAGGCGTTCATTGACTA<br>CTTTGTTAGCACCCTGTATCCGCACGTTCGTC<br>AAGACGAGTTCGTGGATTTTGCGCAGAAACT<br>GTTCCAAGACACCGATGAAATCCGTAGCCTG<br>ACCCTGCTGGCGCTGAGCAGCCAGTATCCGA<br>TTAAGCGTCAAGGCGAGACCGAATAA	
Cas7b	ATGAGCAACCTGAACCTGTTCGCGACCATCC<br>TGACCTATCCGGCGCCGGCGAGCAACTATCG<br>TGGCGAGAGCGAGGAAAACCGTAGCGTGAT<br>CCAGAAGATTCTGAAAGACGGTCAAAAATAC<br>GCGATCATTAGCCCGGAAAGCATGCGTAACG<br>CGCTGCGTGAGATGCTGATTGAACTGGGCCA<br>GCCGAACAACCGTACCCGTCTGCACAGCGA<br>GGACCAACTGGCGGTGGAGTTCAAAGAATA<br>CCCGAACCCGGATAAGTTTGCGGACGATTTC<br>CTGTTTGGTTATATGGTTGCGCAGACCAACG<br>ACGCGAAAGAAATGAAGAAACTGAACCGTC<br>CGGCGAAGCGTGATAGCATCTTCCGTTGCAA	No tag

	CATGGCGGTGGCGGTTAACCCGTACAAATAT GACACCGTGTTTACCAAAGCCCGCTGAACG CGGGTGATAGCGCGTGGAAGAACAGCACCA GCAGCGCGCTGCTGCACCGTGAGGTTACCCA CACCGCGTTCCAGTATCCGTTCGCGCTGGCG GGCAAGGACTGCGCGGCGAAACCGGAGTGG GTGAAGGCGCTGCTGCAAGCGATTGCGGAA CTGAACGGTGTTGCGGGTGGCCATGCGCGTG CGTACTATGAATTTGCGCCGCGTAGCGTGGTT GCGCGTCTGACCCCGAAACTGGTGGCGGGTT ACCAGACCTATGGCTTTGATGCGGAGGGTAA CTGGCTGGAACTGAGCCGTCTGACCGCGACC GACAGCGATAACCTGGACCTGCCGGCGAAC GAGTTTTGGCTGGGTGGCGAACTGGTTCGTA AAATGGATCAGGAGCAAAAGGCGCAACTGG AAGCGATGGGTGCGCACCTGTATGCGAACCC GGAGAAGTTGTTTGCCGACTTAGCAGATAGT TTTCTGGGGGTATAA	
Cas5b	ATGGCGCAGCTGGCGCTGGCGCTGGACACC GTGACCCGTTACCTGCGTCTGAAGGCGCCGT TCGCGGCGTTTCGTCCGTTCCAAAGCGGTAG CTTTCGTAGCACCACCCCGGTGCCGAGCTTC AGCGCGGTTTATGGTCTGCTGCTGAACCTGG CGGGCATCGAGCAGCGTCAAGAGGTGGAGG GTAAAGTTACCCTGATTAAGCCGAAAGCGGA ACTGCCGAAGCTGGCGATCGCGATTGGCCAG GTGAAACCGAGCAGCACCAGCCTGATCAAC CAGCAACTGCACAACTACCCGGTTGGTAACA GCGGCAAGGAGTTTGCGAGCCGTACCTTCGG TAGCAAATATTGGATTGCGCCGGTGCGTCGT GAAGTGCTGGTTAACCTGGACCTGATCATTG GCCTGCAAAGCCCGGTGGAGTTTTGGCAGA AGCTGGATCAAGGTCTGAAAGGCGAAACCG TTATCAACCGTTACGGTCTGCCGTTCGCGGG CGACAACAACTTCCTGTTTGATGAGATCTAC CCGATTGAAAAGCCGGACCTGGCGAGCTGG TATTGCCCGCTGGAGCCGGATACCCGTCCGA ACCAGGGTGCGTGCCGTCTGACCCTGTGGAT CGACCGTGAGAACAACACCCAAACCACCAT TAAGGTTTTTAGCCCGAGCGATTTCCGTCTGG AACCGCCGGCGAAAGCGTGGCAGCAACTGC CGGGCTAA	No tag
Cas6b	ATGAACTTCATCGACCTGGCGTTTCCGGTGA AGGGCACCGTTCTGAACGCGGATCACAACTA	No tag

	CTATCTGTACAGCGCGATTGCGAAAGAGTTT<br>CCGATCCTGCACGACCTGCCGGATCTGGCGG<br>TGAACACCATCAGCGGCAAGCCGGACCGTG<br>AAGGCAAAATTCTGCTGGTTCCGGGCAGCAA<br>GCTGTGGATGCGTCTGCCGATCGATAACATTA<br>CCCACATCTACCAGCTGGCGGGTAAGAAACT<br>GCGTATTGGCCAATATAGCATCGAACTGGGTA<br>ACCCGAGCCTGCACCCGCTGGAGCCGGTTG<br>AAAGCCTGAAGGCGCGTATCATTACCATTAA<br>AGGTCACACCGAGCCGATCAGCTTCCTGGAA<br>GCGGTGAAGCGTCAGCTGTTTGCGCTGGAG<br>ATTACCGAAGGTGACGTTGGCATCCCGGCGA<br>ACCACGAGGGTATTCCGAAACGTCTGACCCT<br>GCAAATCAAGAAACCGGAACGTACCTACAG<br>CATTGTGGGCTATAGCGTTCTGCTGAGCAAC<br>CTGAGCGCGGAGGATAGCCTGAAGATTCAGC<br>AAGTGGGTATCGGTGGCAAACGTCGTCTGGG<br>TTGCGGCGTGTTCTATCCGGCGGTTAAGAAA<br>AGCACCAACAGCGGTAACAAGAAAAACGTT<br>GAAGCGACCCTGGGCTAA	
Cas11b	ATGACCGTGGCGACCACCAAAACCCGTGAG<br>TACGCGGAAATCGTTTATAAGATTGCGCAGG<br>GTTTCGTGCTGAGCAAGCTGAGCAGCAAAC<br>ACGACCTGCAATGGAGCAAGTGCAAAGGCA<br>ACCCGAAACTGGAGCGTGAATACAACGATAA<br>GAAAGAGAAGGTGGTTAACGAAGCGTTTCT<br>GGCGATCCGTAGCCGTACCGAAAAACAGGC<br>GTTCATTGACTACTTTGTTAGCACCCTGTATC<br>CGCACGTTCGTCAAGACGAGTTCGTGGATTT<br>TGCGCAGAAACTGTTCCAAGACACCGATGA<br>AATCCGTAGCCTGACCCTGCTGGCGCTGAGC<br>AGCCAGTATCCGATTAAGCGTCAAGGCGAGA<br>CCGAATAA	No tag
Cas3	CATCACCATCATCACCACAGCCAGCTGGAAG<br>TGCTGTTCCAGGGTCCGGGATCCGGATCCAT<br>GCTGAAACAACTGCTGGCGAAGAGCCTGCC<br>GACCGACCCGCAGAAGAAACCGCTGAGCCT<br>GGAACAACACCTGCTGGATACCGAGACCGC<br>GGCGCTGGTGATCTTTAAGGGTCGTATGCTG<br>GACAACTGGTGCCGTTTCTTTAAGGTTAAAG<br>ACCCGGATGAATTCCTGCTGCACCTGCGTGT<br>GGCGGCGCTGTTTCACGATCTGGGCAAAGCG<br>AACCACGAGTTCATTGAAGCGGTTACCGCGA<br>AAGGTTTTGTGCCGCAGACCCTGCGTCACGA	His_6 tag - PreScission site-Cas3

	ATGGATCAGCGCGCTGGTTCTGCACCTGCCG<br>GAAGTGCGTCAATGGCTGGGCAAAAGCAAC<br>CTGAACCTGGAAGTGGTTACCGCGGCGGTTC<br>TGAGCCATCACCTGAAAGCGAGCCCGGATGG<br>TGATTACAAGTGGGACGAACCGCAGAAGAG<br>CGGTGATAAAGTTGAGACCAAGCTGTATTTC<br>AACCACGAGGAAGTGGACCGTATCCTGAAC<br>AAAATTGCGAACCTGCTGGACGTGGATAGCA<br>AGCTGCCGGAACTGCCGAAGAAATGGATCA<br>AAGGCGACATTTTCCTGGAGAACATCTACAA<br>AGATGCGAACCAGATTGGTCGTAAGTTTACC<br>CGTCAAGCGAAGAAAGACGATAGCCTGAAA<br>GGCCTGCTGCTGGCGGTTAAAGCGGGTCTGA<br>TTGCGAGCGACAGCGTGGCGAGCGGTATTTA<br>CCGTACCCAGGATAGCGAAGCGATCGCGAAC<br>TGGGTTAACCAAACCCTGCACACCAACAGC<br>ATTACCCCGGAGGAAATCGAGGAAAAGATTC<br>TGCACCCGCGTTATCGTCAGGTGGAGAAAAG<br>CATCAACGAACCGTTCCAGCTGAAACGTTTT<br>CAAGAGAAGGCGGAAACCCTGAGCAGCCGT<br>CTGCTGCTGATGAGCGGTTGCGGCAGCGGTA<br>AAACCATTTTCGCGTACAAGTGGATGCAGGG<br>CGTTCTGAACAAGCACCAAGCGGGTCGTGC<br>GATCTTCCTGTATCCGACCCGTGGCACCGCG<br>ACCGAAGGTTTTAAAGACTATGTGAGCTGGT<br>GCCCGGAGGCGGATGCGAGCCTGCTGACCG<br>GTACCGCGACCTACGAGCTGCAGGCGATTGC<br>GAAAAACCCGACCGAGGCGAACGAAGGCAA<br>GGACTATCAAGCGGATGAACGTCTGTACGCG<br>CTGGGCTATTGGGGCAAGCGTTTCTTTAGCG<br>CGACCGTTGACCAGTTCCTGAGCTTTCTGAC<br>CCACAACTACAAAAGCATCTGCCTGCTGCCG<br>GTGCTGGCGGACAGCGTGGTTGTGATCGATG<br>AAATTCACAGCTTCAGCCCGGAGATGTTTGA<br>CAGCCTGGTTTGCTTCCTGAAGACCTTTGAT<br>GTTCCGGTGCTGTGCATGACCGCGACCCTGC<br>CGCAGACCCGTATTGAGGACCTGACCATTCA<br>ACTGGACAAGGATAAAGACGGCCTGGGTCT<br>GGAAGTTTTCCCGACCAGCGATCGTAGCGAG<br>CTGGCGGAGCTGGAAAAAGCGGAGGGCATG<br>GAACGTTACCTGATTGCGCACACCAACGAGG<br>AAGCGGCGCTGGACCTGGCGGTGAAAGCGT<br>ATCAGGATAGCAAGCGTGTTCTGTGGGTTGT<br>GAACACCGTGGACCGTTGCCGTGAGAAGGC	

	GCGTAAACTGGAATGCCTGCTGAAGACCGA GGTTCTGACCTACCACAGCCGTTTCAAACTG GCGGATCGTCAAAACCGTCACCGTGAGACC GTGGAAGCGTTTGCGCTGCACCAGGCGCAA GGTGAAAAGAAAGCGGCGATCGCGGTTACC ACCCAGGTGTGCGAGATGAGCCTGGATCTGG ACGCGGATGTTCTGATCACCGAACTGGCGCC GATTAGCAGCCTGGTGCAACGTTTCGGCCGT AGCAACCGTGGTGACAAGAACGATAAAACC GAGCCGAGCAAAATTTACGTTTATAAGCCGC CGAAGGACAAACCGTATAAGCAGAAAGACG ATCTGGACCCGGCGGAAAAGTTCATCAACGA TGTGCTGGGTCGTGCGAGCCAAAAACTGCTG GCGGAGAAGCTGAAAGAGCATAGCCCGCCG GGCCGTTACAGCGATGGTAGCGCGCCGTTTG TGACCCAGGGCTATTGGGCGAGCAGCGATGA GCCGTTCCGTAAGATTGACGATTTTGCGGTTA ACGCGGTGCTGACCGAGGACCTGGGTGAAA TCACCCAATACCTGAACAGCAACCCGCCGAA ACCGATCGATGGCTTTATTGTTCCGGTGCCGA AGAAATATAAGTTCCAGGGTTTTAGCCACCG TCCGCCGCAACTGCCGAAATACCTGGAAATC GCGGACAGCAAATTCTATAGCAGCAAGCGTG GCTTTGGTGACGATGCG	
RM-1 Forward	CGAAATTAATACGACTCACTATAGGGGAATTG TGAGCGGATAACAATTCCCCTCTAGAAATAAT TTTGTTTAACTTTAAGAAGGAGATATACCAT	The template for RNA Marker1 transcription. The T7 promoter highlighted in green.
RM-1 Reverse	ATGGTATATCTCCTTCTTAAAGTTAAACAAAA TTATTTCTAGAGGGGAATTGTTATCCGCTCAC AATTCCCCTATAGTGAGTCGTATTAATTTCG	The template for RNA Marker1 transcription. The T7 promoter highlighted in green.
RM-2 Forward	CGAAATTAATACGACTCACTATAGGGGAATTG TGAGCGGATAACAATTCCCCTCTAGAAATAAT TTTGTTTAACTTTAAGAAGGAGATATACCATG G	The template for RNA Marker2 transcription. The T7 promoter highlighted in green.
RM-2 Reverse	CCATGGTATATCTCCTTCTTAAAGTTAAACAA AATTATTTCTAGAGGGGAATTGTTATCCGCTC ACAATTCCCCTATAGTGAGTCGTATTAATTTC G	The template for RNA Marker2 transcription. The T7 promoter highlighted in green.
RM-3 Forward	CGAAATTAATACGACTCACTATAGGGGAATTG TGAGCGGATAACAATTCCCCTCTAGAAATAAT TTTGTTTAACTTTAAGAAGGAGATATACCATG GGC	The template for RNA Marker3 transcription. The T7 promoter highlighted in green.

RM-3 Reverse	GCCCATGGTATATCTCCTTCTTAAAGTTAAACAAAATTATTTCTAGAGGGGAATTGTTATCCGCTCACAATTCCCCTATAGTGAGTCGTATTAATTTCG	The template for RNA Marker3 transcription. The T7 promoter highlighted in green.

Supplementary Table 2. Plasmids used in this work

**Name**	**Insert**	**Vector**	**Marker**	**purpose**
pCDF-cas*8b-cas7b-cas5b*	Cas8b-Cas7b-Cas5b	pCDFDuet-1	Streptomycin	purification
pRSF-*cas6b-cas11b*	Cas6b-Cas11b	pRSFDuet-1	Ampicillin	purification
pET-28a-*cas3*	Cas3	pET28a	Kanamycin	purification
pUC19-CRISPR array	CRISPR array	pUC19	Ampicillin	purification
pET-28a-PAM library	PAM library	pET28a	Kanamycin	PAM determination

Supplementary Table 3. Synthetic 59bp PAM library DNA sequences

Name	Sequence (5' to 3')	Note
synPAM-1	TCGCGGATCC**AAA**TTTATCACCGTGTCCCCAATCTGGATA TTTTGTGTCTCGAGCACCA (NTS) TGGTGCTCGAGACACAAAATATCCAGATTGGGGACACGG TGATAAATTTGGATCCGCGA (TS)	AAA PAM-protospacer
synPAM-2	TCGCGGATCC**AAG**TTTATCACCGTGTCCCCAATCTGGATA TTTTGTGTCTCGAGCACCA (NTS) TGGTGCTCGAGACACAAAATATCCAGATTGGGGACACGG TGATAAACTTGGATCCGCGA (TS)	AAG PAM-protospacer
synPAM-3	TCGCGGATCC**AAC**TTTATCACCGTGTCCCCAATCTGGATA TTTTGTGTCTCGAGCACCA (NTS) TGGTGCTCGAGACACAAAATATCCAGATTGGGGACACGG TGATAAAGTTGGATCCGCGA (TS)	AAC PAM-protospacer
synPAM-4	TCGCGGATCC**AAT**TTTATCACCGTGTCCCCAATCTGGATA TTTTGTGTCTCGAGCACCA (NTS) TGGTGCTCGAGACACAAAATATCCAGATTGGGGACACGG TGATAAAATTGGATCCGCGA (TS)	AAT PAM-protospacer
synPAM-5	TCGCGGATCC**AGA**TTTATCACCGTGTCCCCAATCTGGATA TTTTGTGTCTCGAGCACCA (NTS) TGGTGCTCGAGACACAAAATATCCAGATTGGGGACACGG TGATAAATCTGGATCCGCGA (TS)	AGA PAM-protospacer
synPAM-6	TCGCGGATCC**AGG**TTTATCACCGTGTCCCCAATCTGGATA TTTTGTGTCTCGAGCACCA (NTS) TGGTGCTCGAGACACAAAATATCCAGATTGGGGACACGG TGATAAACCTGGATCCGCGA (TS)	AGG PAM-protospacer
synPAM-7	TCGCGGATCC**AGC**TTTATCACCGTGTCCCCAATCTGGATA TTTTGTGTCTCGAGCACCA (NTS) TGGTGCTCGAGACACAAAATATCCAGATTGGGGACACGG TGATAAAGCTGGATCCGCGA (TS)	AGC PAM-protospacer
synPAM-8	TCGCGGATCC**AGT**TTTATCACCGTGTCCCCAATCTGGATA TTTTGTGTCTCGAGCACCA (NTS) TGGTGCTCGAGACACAAAATATCCAGATTGGGGACACGG TGATAAAACTGGATCCGCGA (TS)	AGT PAM-protospacer
synPAM-9	TCGCGGATCC**ACA**TTTATCACCGTGTCCCCAATCTGGATA TTTTGTGTCTCGAGCACCA (NTS) TGGTGCTCGAGACACAAAATATCCAGATTGGGGACACGG TGATAAATGTGGATCCGCGA (TS)	ACA PAM-protospacer
synPAM-10	TCGCGGATCC**ACG**TTTATCACCGTGTCCCCAATCTGGATA TTTTGTGTCTCGAGCACCA (NTS) TGGTGCTCGAGACACAAAATATCCAGATTGGGGACACGG TGATAAACGTGGATCCGCGA (TS)	ACG PAM-protospacer

synPAM-11	TCGCGGATCC**ACC**TTTATCACCGTGTCCCCAATCTGGATATTTTGTGTCTCGAGCACCA (NTS) TGGTGCTCGAGACACAAAATATCCAGATTGGGGACACGGTGATAAAGGTGGATCCGCGA (TS)	ACC PAM-protospacer
synPAM-12	TCGCGGATCC**ACT**TTTATCACCGTGTCCCCAATCTGGATATTTTGTGTCTCGAGCACCA (NTS) TGGTGCTCGAGACACAAAATATCCAGATTGGGGACACGGTGATAAAAGTGGATCCGCGA (TS)	ACT PAM-protospacer
synPAM-13	TCGCGGATCC**ATA**TTTATCACCGTGTCCCCAATCTGGATATTTTGTGTCTCGAGCACCA (NTS) TGGTGCTCGAGACACAAAATATCCAGATTGGGGACACGGTGATAAATATGGATCCGCGA (TS)	ATA PAM-protospacer
synPAM-14	TCGCGGATCC**ATG**TTTATCACCGTGTCCCCAATCTGGATATTTTGTGTCTCGAGCACCA (NTS) TGGTGCTCGAGACACAAAATATCCAGATTGGGGACACGGTGATAAACATGGATCCGCGA (TS)	ATG PAM-protospacer
synPAM-15	TCGCGGATCC**ATC**TTTATCACCGTGTCCCCAATCTGGATATTTTGTGTCTCGAGCACCA (NTS) TGGTGCTCGAGACACAAAATATCCAGATTGGGGACACGGTGATAAAGATGGATCCGCGA (TS)	ATC PAM-protospacer
synPAM-16	TCGCGGATCC**ATT**TTTATCACCGTGTCCCCAATCTGGATATTTTGTGTCTCGAGCACCA (NTS) TGGTGCTCGAGACACAAAATATCCAGATTGGGGACACGGTGATAAAAATGGATCCGCGA (TS)	ATT PAM-protospacer
synPAM-17	TCGCGGATCC**TTG**TTTATCACCGTGTCCCCAATCTGGATATTTTGTGTCTCGAGCACCA (NTS) TGGTGCTCGAGACACAAAATATCCAGATTGGGGACACGGTGATAAACAAGGATCCGCGA (TS)	TTG PAM-protospacer
synPAM-18	TCGCGGATCC**CTG**TTTATCACCGTGTCCCCAATCTGGATATTTTGTGTCTCGAGCACCA (NTS) TGGTGCTCGAGACACAAAATATCCAGATTGGGGACACGGTGATAAACAGGGATCCGCGA (TS)	CTG PAM-protospacer
synPAM-19	TCGCGGATCC**GTG**TTTATCACCGTGTCCCCAATCTGGATATTTTGTGTCTCGAGCACCA (NTS) TGGTGCTCGAGACACAAAATATCCAGATTGGGGACACGGTGATAAACACGGATCCGCGA (TS)	GTG PAM-protospacer
synPAM-20	TCGCGGATCC**TCG**TTTATCACCGTGTCCCCAATCTGGATATTTTGTGTCTCGAGCACCA (NTS) TGGTGCTCGAGACACAAAATATCCAGATTGGGGACACGGTGATAAACGAGGATCCGCGA (TS)	TCG PAM-protospacer
synPAM-21	TCGCGGATCC**GCG**TTTATCACCGTGTCCCCAATCTGGATATTTTGTGTCTCGAGCACCA (NTS) TGGTGCTCGAGACACAAAATATCCAGATTGGGGACACGGTGATAAACGCGGATCCGCGA (TS)	GCG PAM-protospacer

synPAM-22	TCGCGGATCC**CCG**TTTATCACCGTGTCCCCAATCTGGATATTTTGTGTCTCGAGCACCA (NTS)<br>TGGTGCTCGAGACACAAAATATCCAGATTGGGGACACGGTGATAAACGGGGATCCGCGA (TS)	CCG PAM-protospacer

Supplementary Table 4. Cryo-EM data collection, refinement and validation statistics

	Partial R-loop (EMDB-34495) (PDB 8H67)	Full R-loop (EMDB-35629) (PDB 8IP0)
**Data collection and processing**		
Magnification	130k	130k
Voltage (kV)	300	300
Electron exposure (e–/Å^2)	50	50
Defocus range (μm)	1-2.1	1-2.1
Pixel size (Å)	1.1	1.1
Symmetry imposed	C1	C1
Initial particle images (no.)	667380	667380
Final particle images (no.)	33011	92442
Map resolution (Å)	3.84	3.6
FSC threshold	0.143	0.143
Map resolution range (Å)	N/A	N/A
**Refinement**		
Initial model used (PDB code)	AlphaFold 2	AlphaFold 2
Model resolution (Å)	4.5	4.3
FSC threshold	0.5	0.5
Model resolution range (Å)	N/A	N/A
Map sharpening *B* factor (Å^2)	0	0
Model composition		
Non-hydrogen atoms	25622	26526
Protein residues/Nucleotide	3050/58	3030/110
Ligands	0	0
B factors (Å^2)		
Protein	109.89/470.16/196.88	30.00/423.02/174.85
Nucleotide	20.00/317.14/184.20	20/332.34/184.39
R.m.s. deviations		
Bond lengths (Å)	0.003	0.003
Bond angles (°)	0.689	0.671
Validation		
MolProbity score	2.34	2.30
Clashscore	19.75	18.58
Poor rotamers (%)	0.08	0.43
Ramachandran plot		
Favored (%)	90.14	90.67
Allowed (%)	9.63	9.13
Disallowed (%)	0.23	0.20

Supplementary Table 5. Pacbio sequencing results from *Syn-TRAC*-G1 Cascade + Cas3 edited CD3+ cells

Deletion start positions are relative to PAM site (ATG, +1)

**Number**	**Deletion Start**	**Deletion Size (bp)**	**Note**
1	328	4585	deletion
2	328	4585	deletion
3	328	4585	deletion
4	328	4585	deletion
5	328	4585	deletion
6	324	4585	deletion
7	-85	3577	deletion
8	345	3276	deletion
9	345	3276	deletion
10	329	2787	deletion
11	309	2739	deletion
12	330	2542	deletion
13	302	2220	deletion
14	332	2216	deletion
15	332	2216	deletion
16	303	2056	deletion
17	343	1887	deletion
18	343	1887	deletion
19	343	1887	deletion
20	335	1710	deletion
21	341	1374	deletion
22	331	1193	deletion
23	337	906	deletion
24	337	906	deletion
25	345	788	deletion
26	346	713	deletion
27	345	708	deletion
28	335	673	deletion
29	305	484	deletion
30	326	416	deletion
31	337	364	deletion
32	337	364	deletion
33	337	364	deletion
34	341	289	deletion
35	339	262	deletion
36	344	242	deletion
37	342	218	deletion
38	340	190	deletion
39	340	184	deletion

40	340	184	deletion
41	343	115	deletion
42	344	4078	insertion
43	343	3531	insertion
44	344	3339	insertion
45	342	2193	insertion
46	342	2191	insertion
47	343	2084	insertion
48	343	2083	insertion
49	343	1372	insertion
50	346	1275	insertion
51	345	1105	insertion
52	344	846	insertion
53	344	629	insertion
54	343	580	insertion
55	344	557	insertion
56	344	524	insertion
57	343	445	insertion
58	344	412	insertion
59	345	411	insertion
60	339	352	insertion
61	344	351	insertion
62	344	351	insertion
63	344	347	insertion
64	345	342	insertion
65	345	334	insertion
66	345	334	insertion
67	344	258	insertion
68	348	255	insertion
69	344	254	insertion
70	344	233	insertion
71	340	197	insertion
72	345	192	insertion
73	339	114	insertion

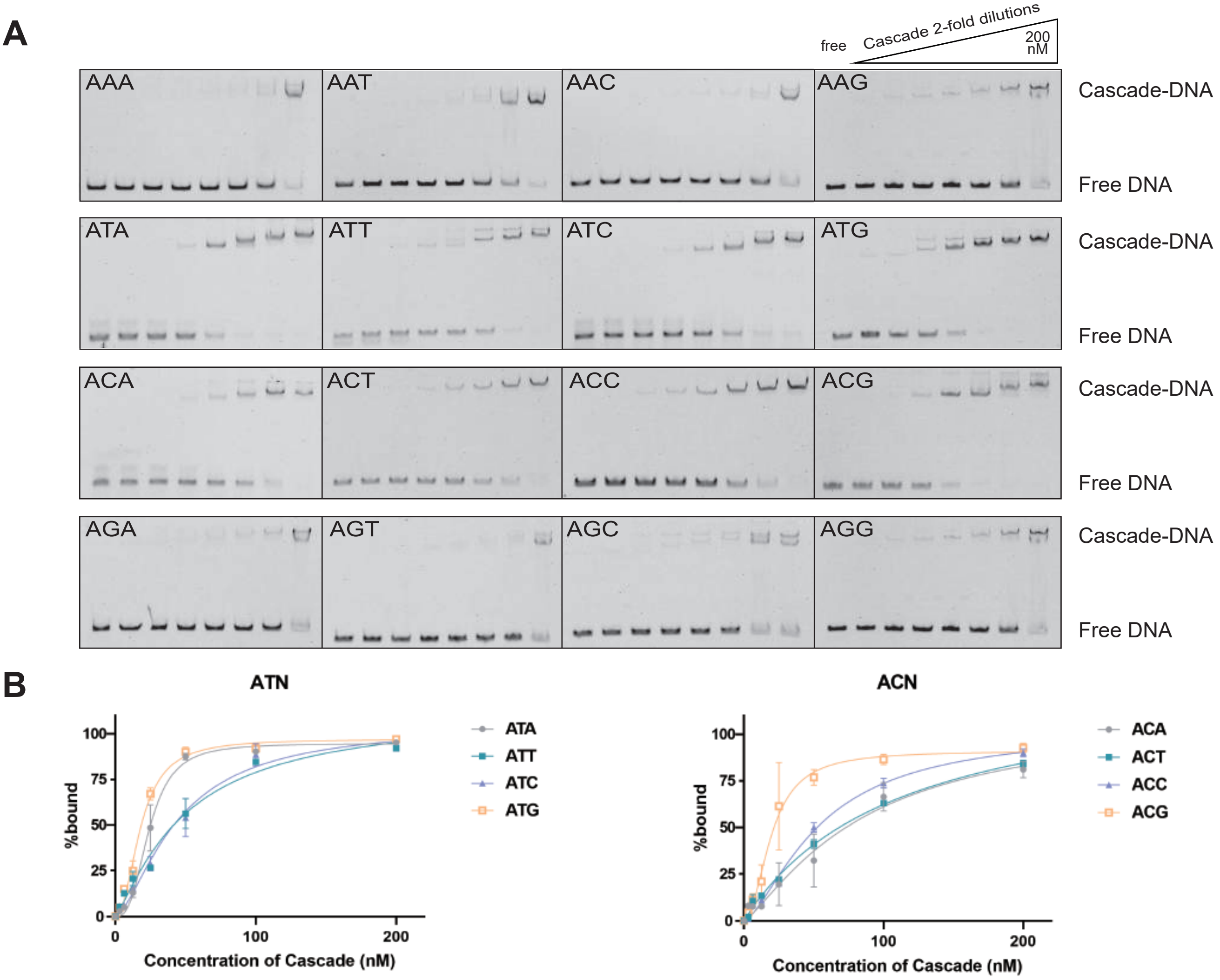

PAM sequence	ATA	ATT	ATC	ATG	ACA	ACT	ACC	ACG
K_d (nM)	24.0±2.1	59.7±23.3	47.7±11.3	18.0±1.7	56.2±11.2	139.9±61.8	58.2±10.6	20.6±4.7

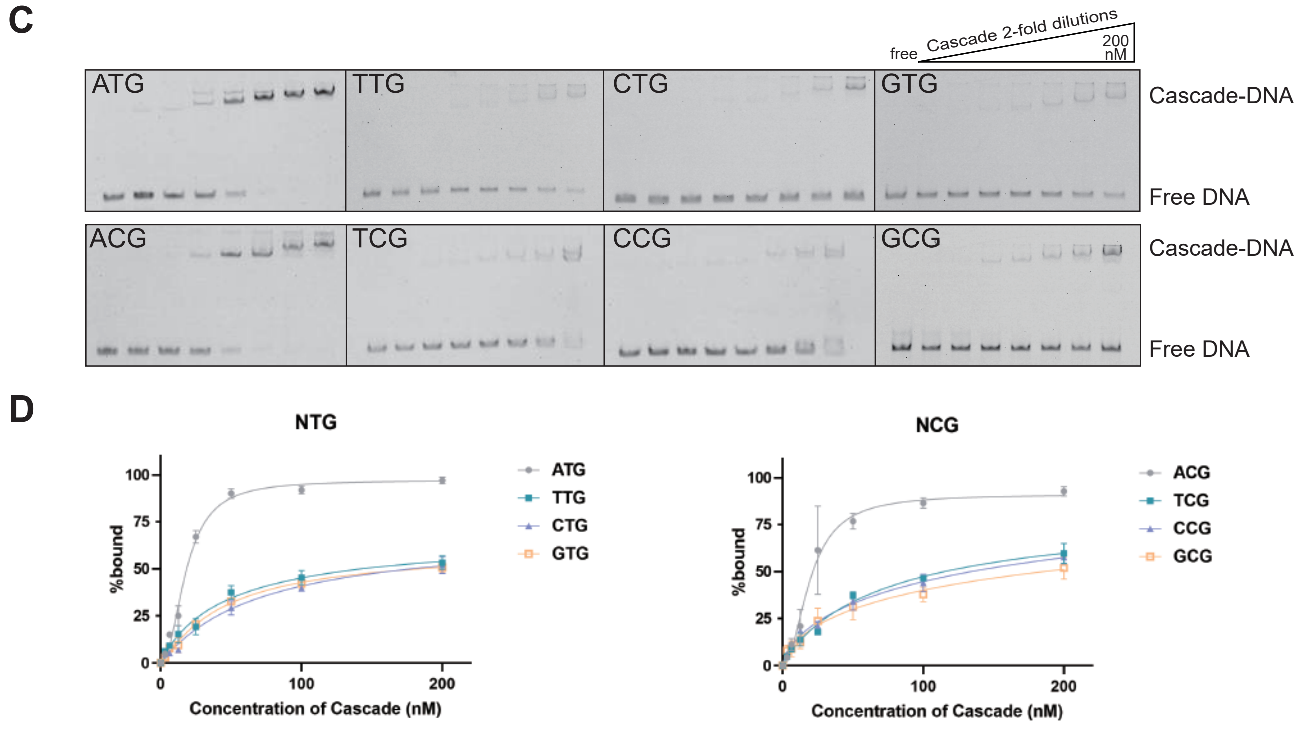

Supplementary Figure 1. PAM identification by EMSA.
(A) EMSA measured the binding of increasing concentrations of Cascade (0-200 nM) to fluorescently labeled dsDNA target with a 5'-ANN PAM (10 nM). (B) Quantification of EMSA data of 5'-ATN and 5'-ACN PAM. Each point is the average of three independent replicates. (C) EMSA verified the preference of -3 position in PAM when the -2 and -1 position fixed as Y and G. (D) The 5'-AYG PAM shown the highest preference.

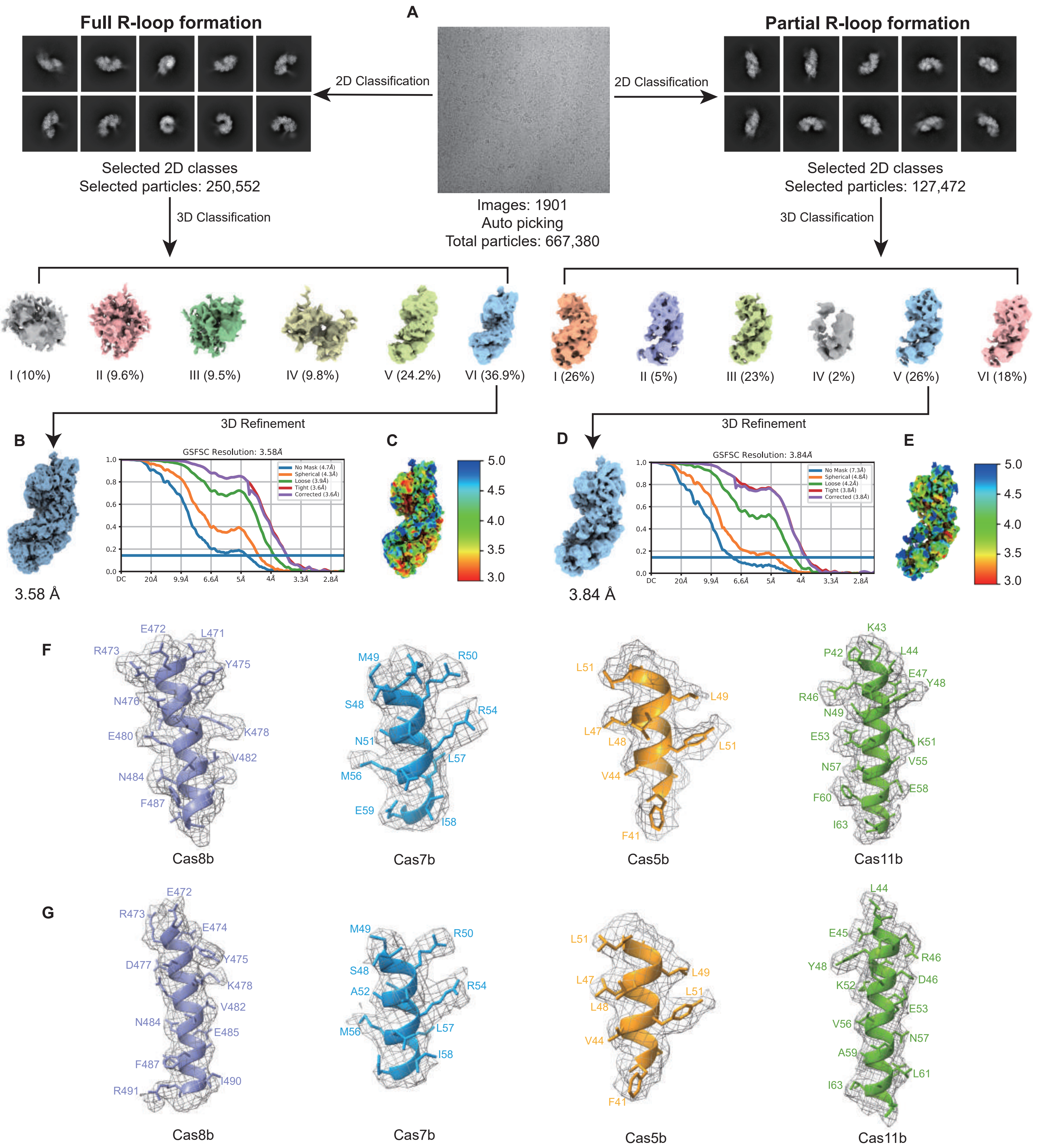

Supplementary Figure 2. Flow-chart of the cryoEM single particle reconstructions of *Syn* Cascade-Target DNA.

(A) Data processing workflow for type I-B Cascade-Target DNA reconstruction. (B) 3.58 Å resolution cryo-EM density (left) and FSC plot (right) of the type I-B Cascade-Target DNA in full R-loop formation. (C) 3.58 Å resolution cryo-EM density colored by local resolution. (D) 3.84 Å resolution cryo-EM density (left) and FSC plot (right) of the type I-B Cascade-Target DNA in partial R-loop formation. (E) 3.84 Å resolution cryo-EM density colored by local resolution. (F) Representative EM densities for Cas8b , Cas7b, Cas5b and Cas11b, superimposed with their corresponding structural model in full R-loop state. (G) Representative EM densities for Cas8b , Cas7b, Cas5b and Cas11b, superimposed with their corresponding structural model in partial R-loop state.

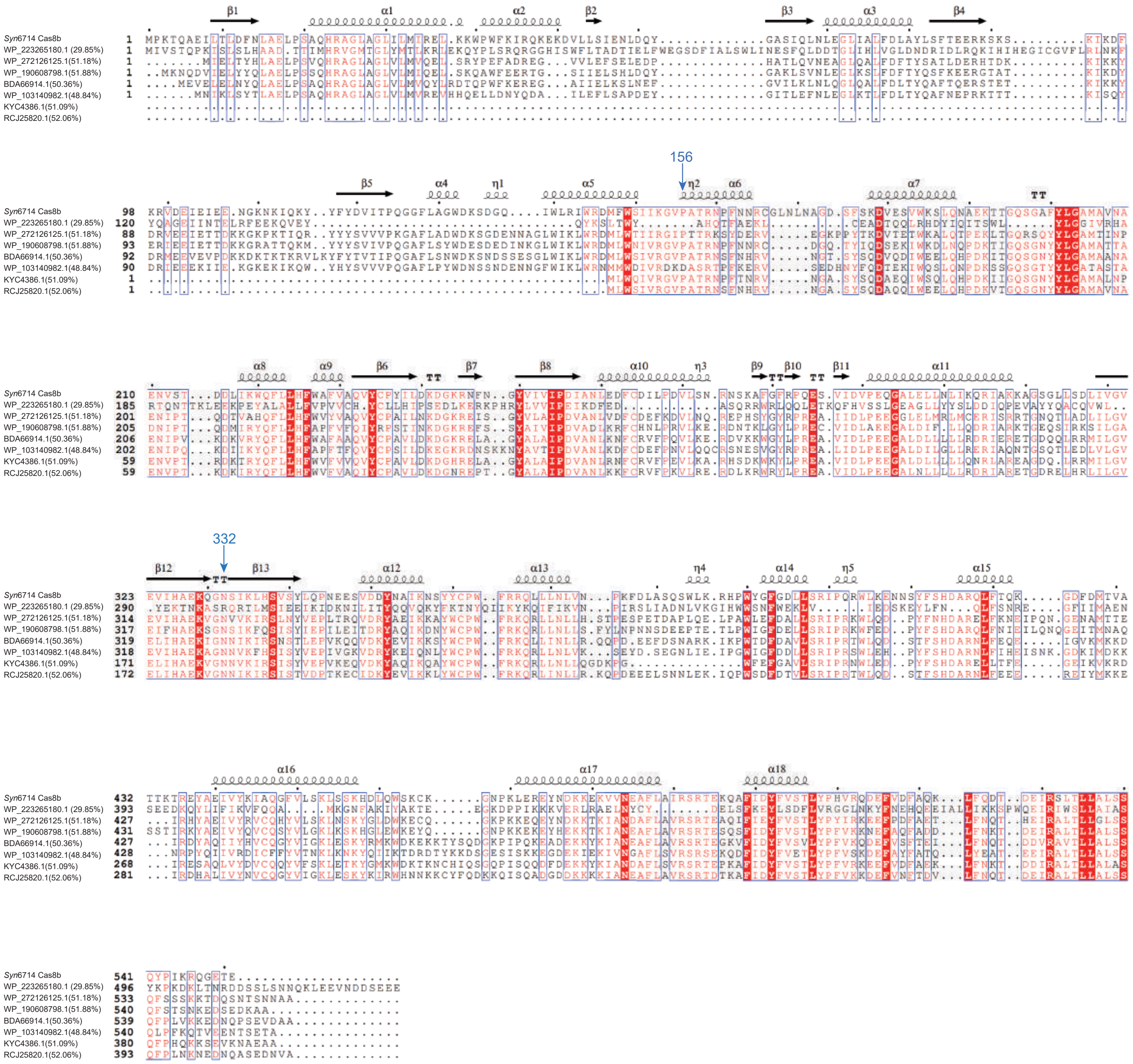

Supplementary Figure 3. Multiple sequence alignment of *Syn*Cas8b and other large subunits of type I-B CRISPR system.

*Syn*Cas8b shares very low sequence homology with the large subunits in Type I systems. Its highest homology with large subunits of the same subtype is approximately 52%, and it has about 30% homology with the large subunit in the Type I-B CAST system. The symbols of a helix, arrow, and letter T denote the secondary structures as α-helix, β-sheet, and β-turn, respectively.

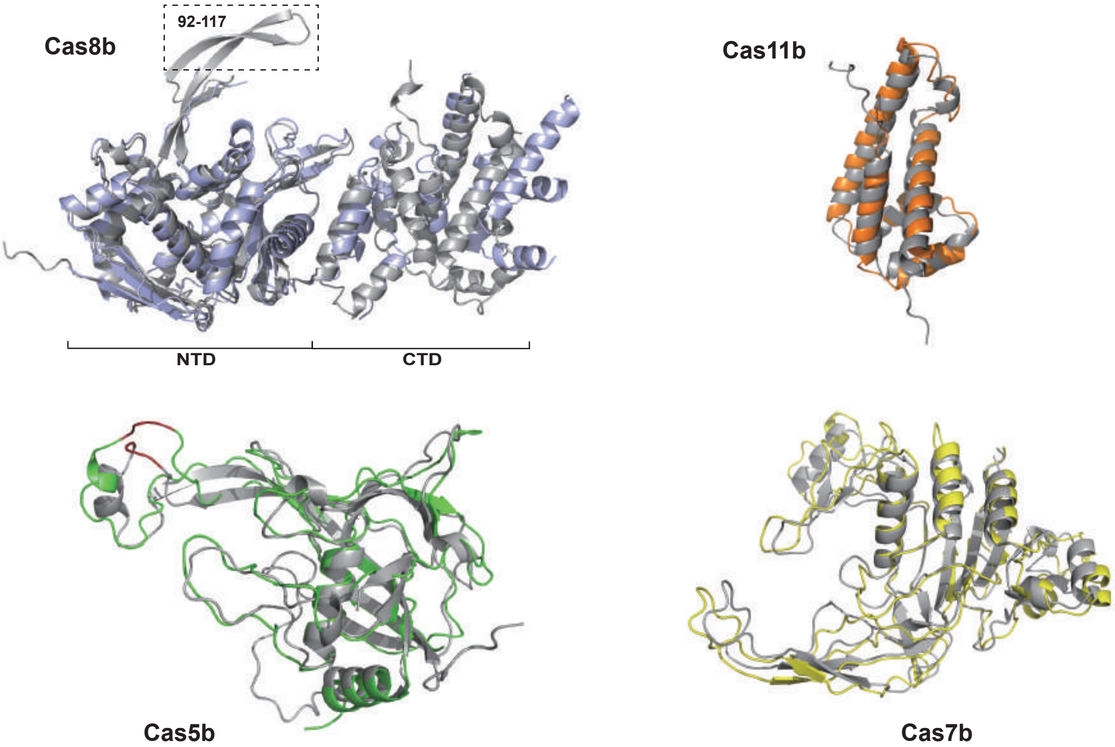

Supplementary Figure 4. The alignment of final modeled and predicted Cas proteins.
The large subunit was modeled with the exception of the β-sheet, which is indicated by a dashed line. For the other modeled Cas proteins, all secondary structures were successfully modeled.

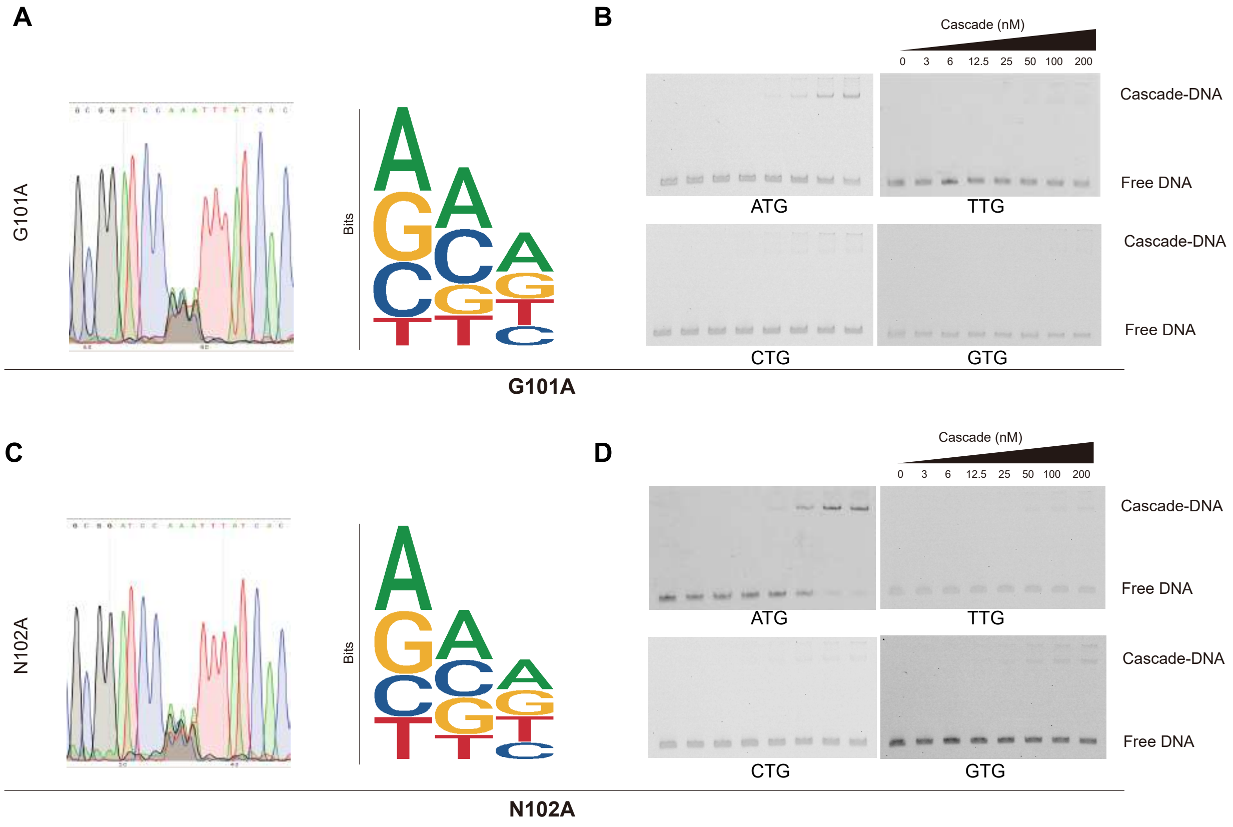

Supplementary Figure 5. Mutagenesis EMSA verified the involvement of G101 and N102 of Cas5b in PAM recognition.

(A) Sanger sequencing of the captured DNA by G101A Cascade from the PAM library using EMSA. (B) The EMSA of 5'-NTG PAM with G101A Cascade. (C) Sanger sequencing of the captured DNA by N102A Cascade from the PAM library using EMSA. (D) The EMSA of 5'-NTG PAM with N102A Cascade.

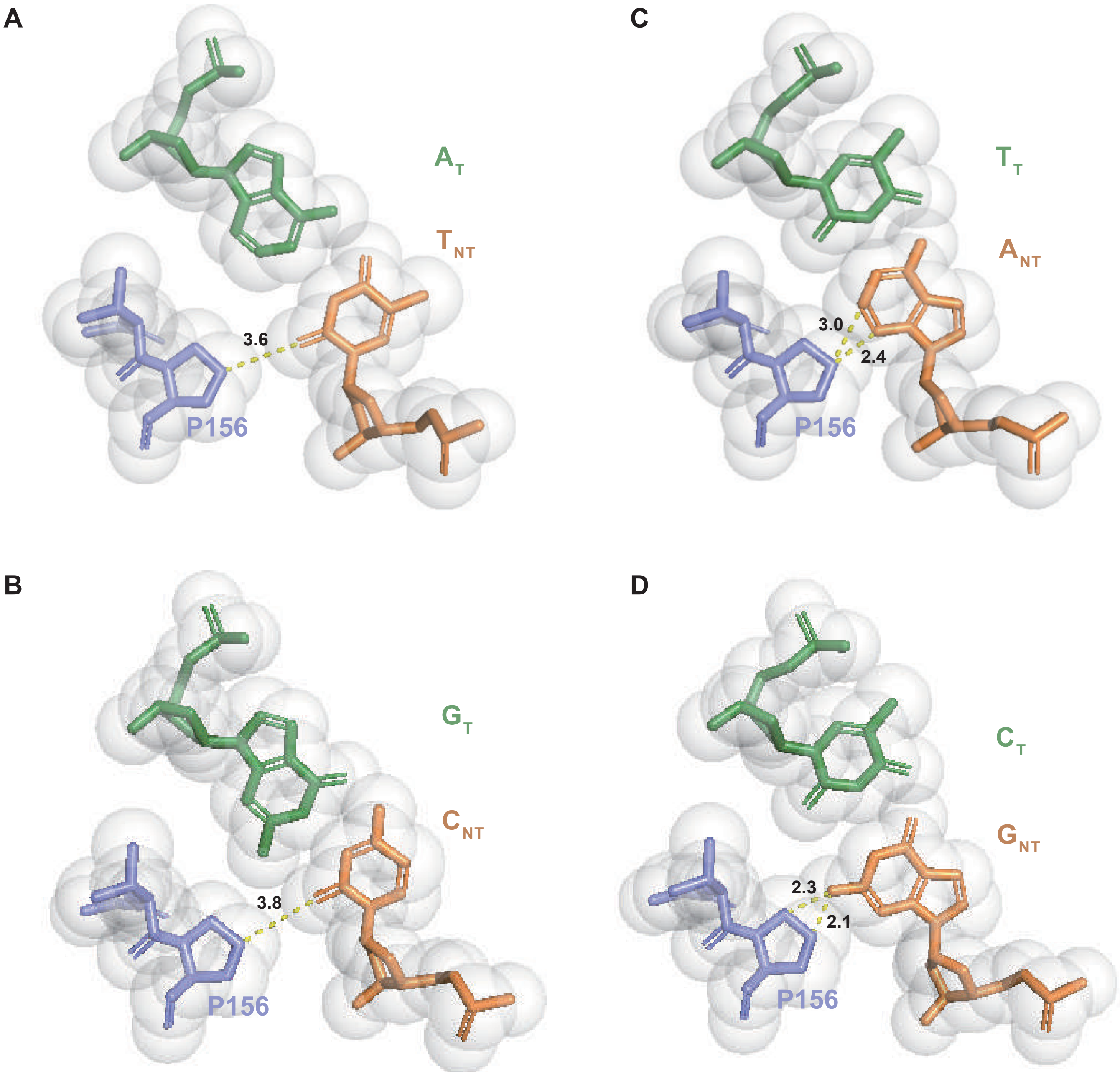

Supplementary Figure 6. Rationalization of PAM -2 specificity using nucleotide substitution and modelling.

Modelling of alternative base pairs at PAM -2 suggests that G_{NT-2} and A_{NT-2} would cause steric clashes with P156 of Cas8b. (A) interaction pattern of Cas8b and PAM-2 in our modelled structure. (B) the initial T_{NT}-A_T pair is substituted as C_{NT}-G_T. The yellow dashes indicate the distances between Cγ of P156 and carbonyl oxygen of the substituted C_{NT-2}. (C) the initial T_{NT}-A_T pair is substituted as A_{NT}-T_T. The yellow dash means the distance between Cγ of P156 and N2/N3 of the substituted A_{NT-2}. (D) the initial T_{NT}-A_T pair is substituted as G_{NT}-C_T. The yellow dashes indicate the distance between Cβ/Cγ of P156 and the amino group of the substituted G_{NT-2}.

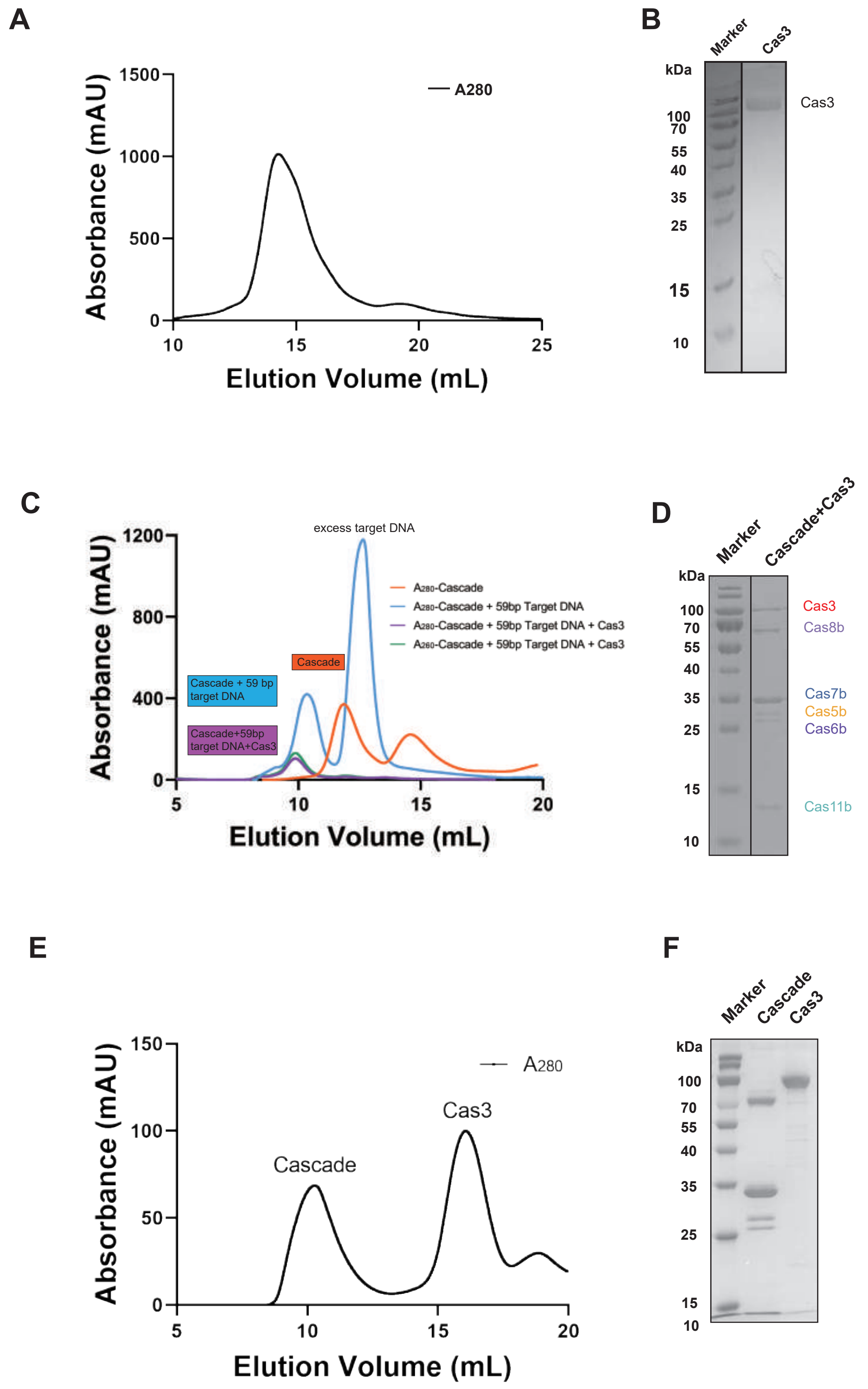

Supplementary Figure 7. Validation of the Cas3 recruitment mode in type I-B CRISPR system
(A) The SEC chromatogram of recombinant Cas3 purified via an N-terminal 6His-tag. (B) Coomassie blue stained SDS-PAGE gel of Cas3 collected at ~ 14.2 ml elution volume in A. (C) Cas3 was incubated with Cascade-dsDNA and then was validated using SEC. The overlay of the SEC chromatograms of the Cascade, Cascade-dsDNA and Cascade-dsDNA-Cas3 complex. (D) Coomassie blue stained SDS-PAGE gel of the fraction peak indicated as Cascade-dsDNA-Cas3 complex. (E) Cas3 was incubated with Cascade, and the sample was loaded and validated by SEC. (F) Coomassie blue stained SDS-PAGE gel of each fraction peak indicated that Cas3 couldn't bind with apo-Cascade.

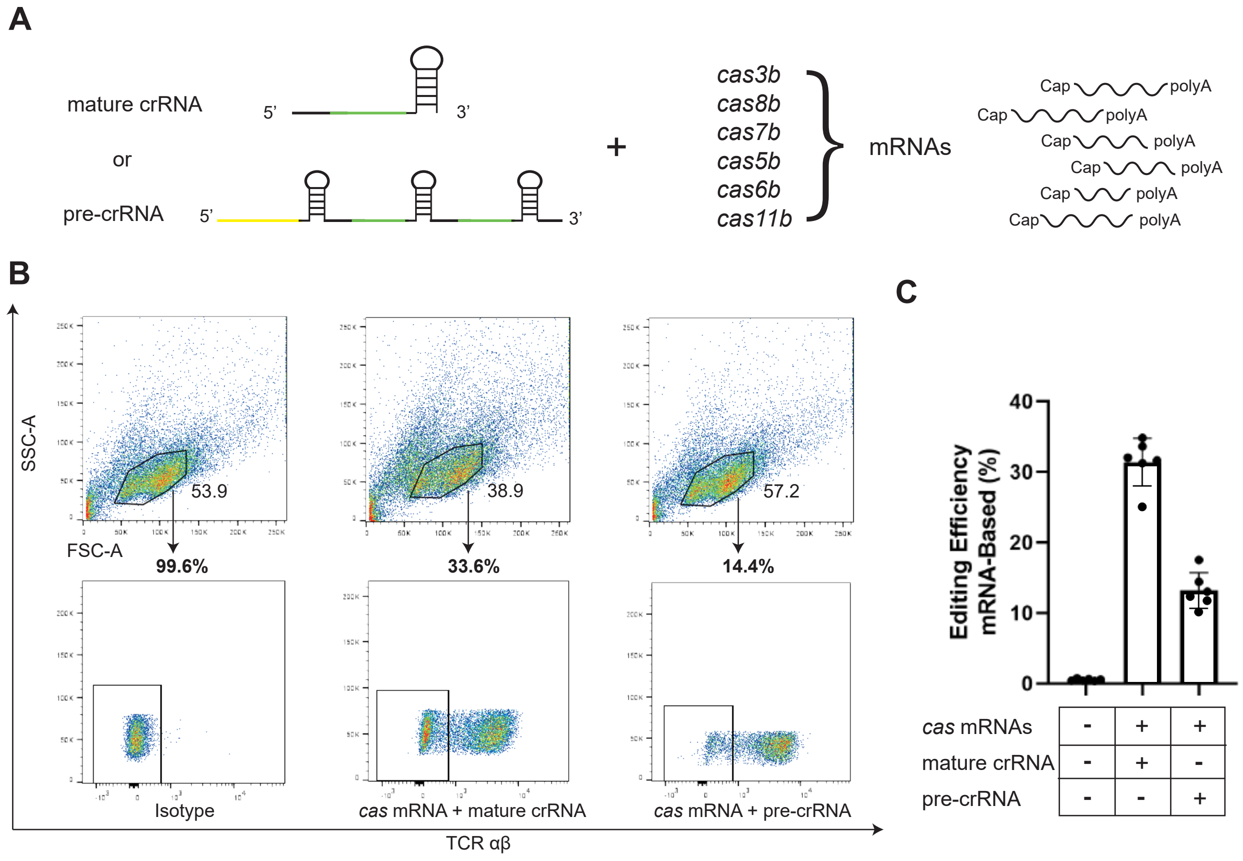

Supplementary Figure 8. The comparison of genome editing efficiency using crRNA of different type. (A) Schematics of cas mRNAs and different crRNA used for genome editing in T cells. The TRAC-targeting CRISPR spacer shown in green. The RNAs were electroporated into T cells. (B) Representative flow cytometry plots of experiment, with percentages of TCR- T cells in the population shown on the top. (C) Editing efficiencies were evaluated and plotted. Data are shown as mean ± SEM, n=6 independent healthy donors.

A

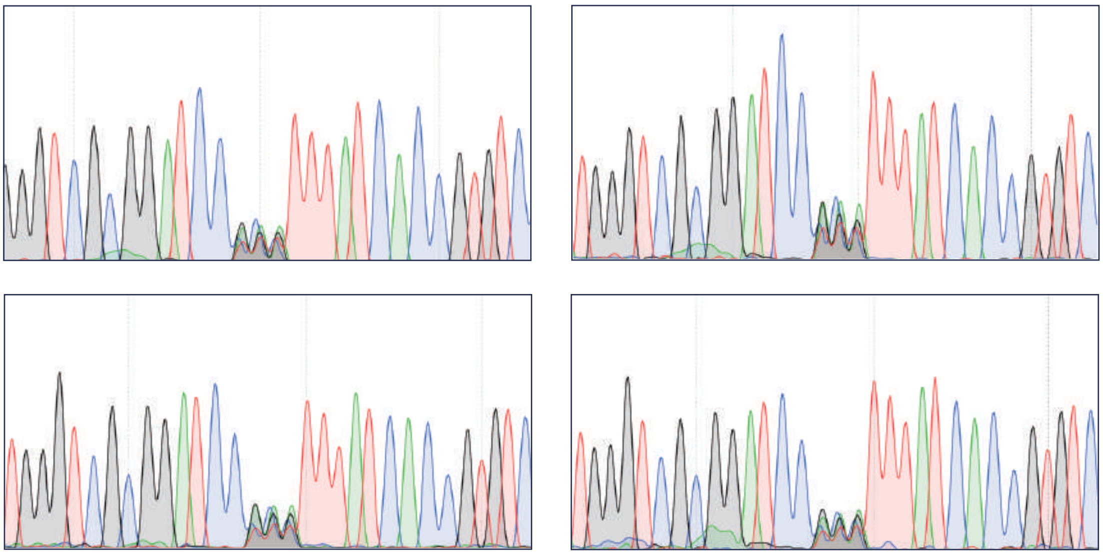

B

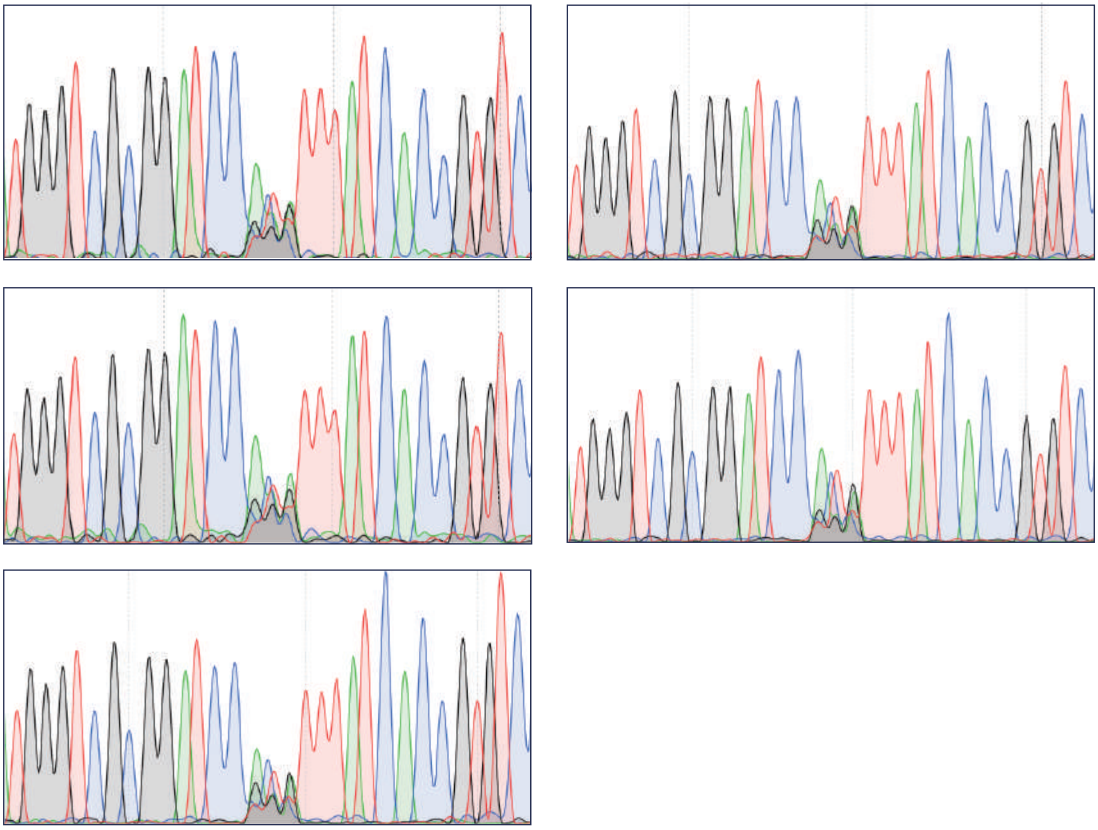

Supplementary Figure 9. Sanger sequencing images for PAM identification
(A) Sanger Sequencing images of the constructed PAM library. Four replicates were performed. (B) Sanger sequencing images of PCR-amplified products from Cascade-bound DNA. Five replicates were performed.

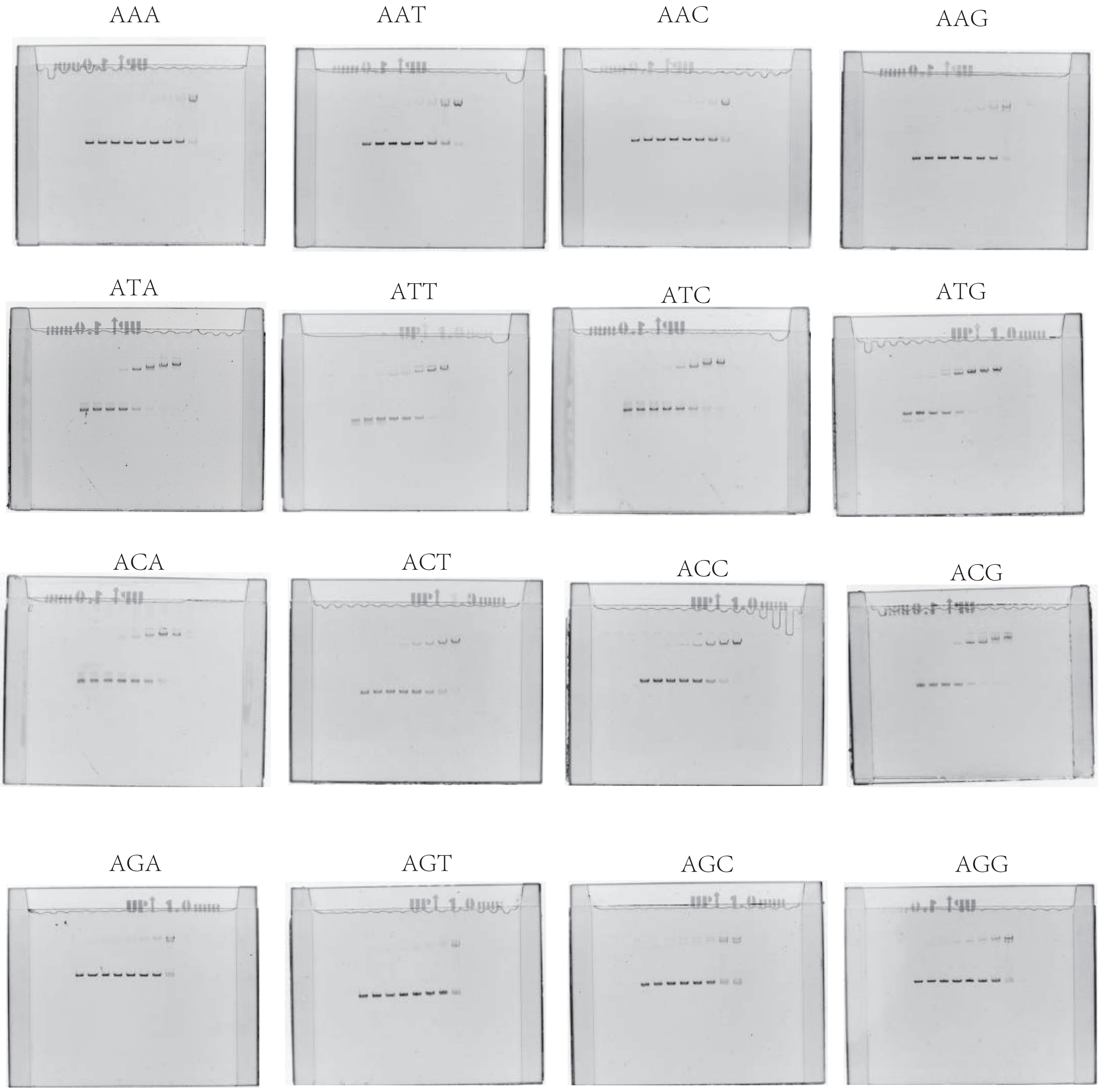

Figure 1A. Uncropped EMSA gel

ATG TTG CTG GTG

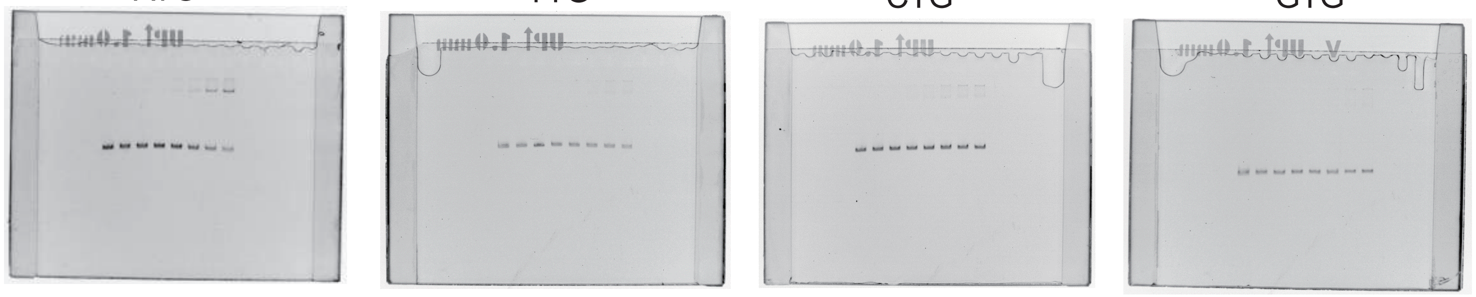

Figure S5B uncropped EMSA gel

ATG TTG CTG GTG

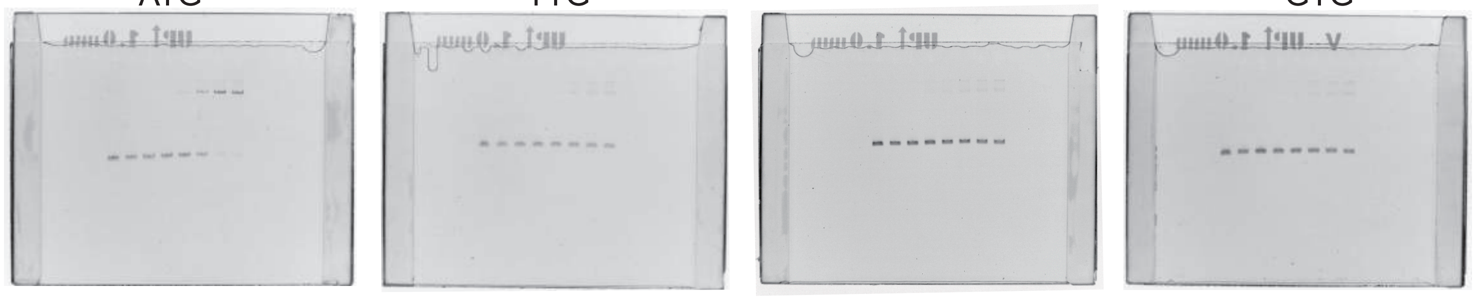

Figure S5D uncropped EMSA gel

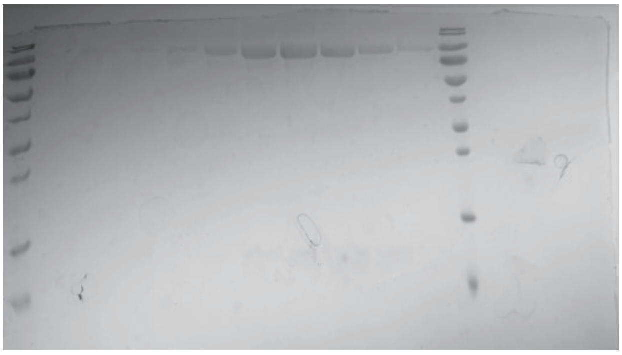

Figure S7B uncropped SDS-PAGE

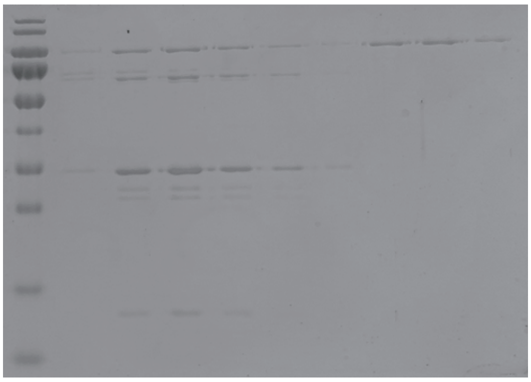

Figure S7D uncropped SDS-PAGE

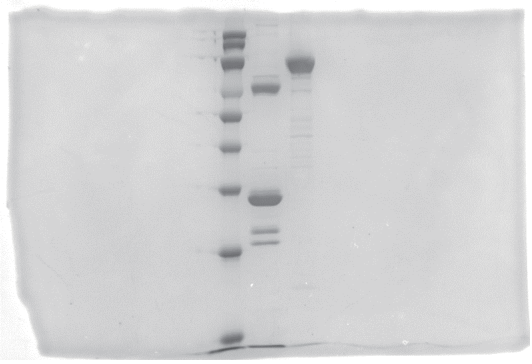

Figure S7F uncropped SDS-PAGE
