# Supplementary material for: Structure and genome editing of type I-B CRISPR-Cas
Source: Nat Commun. 2024 May 15;15:4126. doi: 10.1038/s41467-024-48598-2 (PMC11096372; doi:10.1038/s41467-024-48598-2)
Supplement: Supplementary file 2 — Reporting Summary [file 41467_2024_48598_MOESM2_ESM.pdf]

nature portfolio

Corresponding author(s): Meiling Lu, Zhenhuang Yang, Yibei Xiao

Last updated by author(s): Apr 26, 2024

Reporting Summary

Nature Portfolio wishes to improve the reproducibility of the work that we publish. This form provides structure for consistency and transparency in reporting. For further information on Nature Portfolio policies, see our Editorial Policies and the Editorial Policy Checklist.

Statistics

For all statistical analyses, confirm that the following items are present in the figure legend, table legend, main text, or Methods section.

n/a	Confirmed	
☐	☒	The exact sample size (*n*) for each experimental group/condition, given as a discrete number and unit of measurement
☐	☒	A statement on whether measurements were taken from distinct samples or whether the same sample was measured repeatedly
☒	☐	The statistical test(s) used AND whether they are one- or two-sided<br>*Only common tests should be described solely by name; describe more complex techniques in the Methods section.*
☒	☐	A description of all covariates tested
☒	☐	A description of any assumptions or corrections, such as tests of normality and adjustment for multiple comparisons
☐	☒	A full description of the statistical parameters including central tendency (e.g. means) or other basic estimates (e.g. regression coefficient) AND variation (e.g. standard deviation) or associated estimates of uncertainty (e.g. confidence intervals)
☒	☐	For null hypothesis testing, the test statistic (e.g. *F*, *t*, *r*) with confidence intervals, effect sizes, degrees of freedom and *P* value noted<br>*Give P values as exact values whenever suitable.*
☒	☐	For Bayesian analysis, information on the choice of priors and Markov chain Monte Carlo settings
☒	☐	For hierarchical and complex designs, identification of the appropriate level for tests and full reporting of outcomes
☒	☐	Estimates of effect sizes (e.g. Cohen's *d*, Pearson's *r*), indicating how they were calculated

Our web collection on statistics for biologists contains articles on many of the points above.

Software and code

Policy information about availability of computer code

Data collection	SerialEM in Titan Krios was used for cryo-EM movies collection. LSR Fortessa (BD) was used for flow cytometry data collection.
Data analysis	cryoSPARC v.4.2, UCSF ChimeraX 1.5, AlphaFold2, Coot 0.9.4, Phenix 1.20.1-4487, ESPript 3.0. Pymol 2.5, FlowJo v10.7.1, GraphPad Prism 8.0.1

For manuscripts utilizing custom algorithms or software that are central to the research but not yet described in published literature, software must be made available to editors and reviewers. We strongly encourage code deposition in a community repository (e.g. GitHub). See the Nature Portfolio guidelines for submitting code & software for further information.

Data

Policy information about availability of data

All manuscripts must include a data availability statement. This statement should provide the following information, where applicable:

- Accession codes, unique identifiers, or web links for publicly available datasets
- A description of any restrictions on data availability
- For clinical datasets or third party data, please ensure that the statement adheres to our policy

The cryo-EM reconstructed density map of Syn Cascade-dsDNA complex in partial and full R-loop formations have been deposited into Electron Microscopy data bank under accession number EMD-34495 and EMD-35629, respectively. The associated atomic coordinates have been deposited into Protein Data Bank with PDB code 8H67 and 8IP0.

Research involving human participants, their data, or biological material

Policy information about studies with human participants or human data. See also policy information about sex, gender (identity/presentation), and sexual orientation and race, ethnicity and racism.

Reporting on sex and gender	N/A
Reporting on race, ethnicity, or other socially relevant groupings	N/A
Population characteristics	N/A
Recruitment	N/A
Ethics oversight	N/A

Note that full information on the approval of the study protocol must also be provided in the manuscript.

Field-specific reporting

Please select the one below that is the best fit for your research. If you are not sure, read the appropriate sections before making your selection.

☒ Life sciences ☐ Behavioural & social sciences ☐ Ecological, evolutionary & environmental sciences

For a reference copy of the document with all sections, see nature.com/documents/nr-reporting-summary-flat.pdf

Life sciences study design

All studies must disclose on these points even when the disclosure is negative.

Sample size	The number of particles needed was determined by the resolution of the structures required to support the claims of this study.
Data exclusions	No data was excluded.
Replication	For all genome editing experiments were repeated in at least five independent healthy human donors with successful replication. The EMSA experiments for binding affinity quantification were repeated at least three independent experiments with successful replication.
Randomization	Particles begin in randomized orientations during data processing and are automatically picked using cryoSPARC (version 4.2).
Blinding	No blinding was performed, as the data presented does not require blinding to avoid bias.

Reporting for specific materials, systems and methods

We require information from authors about some types of materials, experimental systems and methods used in many studies. Here, indicate whether each material, system or method listed is relevant to your study. If you are not sure if a list item applies to your research, read the appropriate section before selecting a response.

Materials & experimental systems

n/a	Involved in the study	
☐	☒	Antibodies
☐	☒	Eukaryotic cell lines
☒	☐	Palaeontology and archaeology
☒	☐	Animals and other organisms
☒	☐	Clinical data
☒	☐	Dual use research of concern
☒	☐	Plants

Methods

n/a	Involved in the study	
☒	☐	ChIP-seq
☐	☒	Flow cytometry
☒	☐	MRI-based neuroimaging

Antibodies

Antibodies used	Target- Clone- Vendor- Catolog Number<br>TCR αβ- IP26- BioLegend- 306717

	IgG1-QA16A12- BioLegend- 403505
Validation	Antibody validations were performed by antibody suppliers per quality assurance literature provided by each supplier.

Eukaryotic cell lines

Policy information about cell lines and Sex and Gender in Research

Cell line source(s)	Peripheral blood mononuclear cells were obtained from healthy human donors from StemCell Technologies.
Authentication	CD3+ T cells were isolated by magnetic negative selection using an EasySep Human T Cell. T cells were cultured in Gibco CTS AIM V Medium (Thermo Fisher) and stimulated for 2 days with anti-human CD3/CD28 magnetic dynabeads (Thermo Fisher) at a beads to cells concentration of 1:1 supplemented with human IL-2 at 200 U/ml(Peprotech, New Jersey, USA).
Mycoplasma contamination	Cell lines were not tested for Mycoplasma contaminatin.
Commonly misidentified lines (See ICLAC register)	No commonly misidentified lines were used.

Plants

Seed stocks	No seed was used in our study.
Novel plant genotypes	No seed was used in our study.
Authentication	No seed was used in our study.

Flow Cytometry

Plots

Confirm that:

- ☒ The axis labels state the marker and fluorochrome used (e.g. CD4-FITC).
- ☒ The axis scales are clearly visible. Include numbers along axes only for bottom left plot of group (a 'group' is an analysis of identical markers).
- ☒ All plots are contour plots with outliers or pseudocolor plots.
- ☒ A numerical value for number of cells or percentage (with statistics) is provided.

Methodology

Sample preparation	Peripheral blood mononuclear cells were obtained from healthy human donors from StemCell Technologies. CD3+ T cells were then further isolated by magnetic negative selection using an EasySep Human T Cell. Immediately after isolation, T cells were cultured in Gibco CTS AIM V Medium (Thermo Fisher) and stimulated for 2 days with anti-human CD3/CD28 magnetic dynabeads at the beads to cells concentration ratio of 1:1 supplemented with human IL-2 at 200 U/ml. After electroporation, T cells were cultured in media with IL-2 at 100 U/ml. Every 2-3 days after electroporation, additional media was added, along with additional fresh IL-2 to bring the final concentration to 100 U/ml.
Instrument	LSR Fortessa (BD)
Software	BD FACSDiva™ Software
Cell population abundance	Approximately 10,000 events were acquired for each sample.
Gating strategy	FSC/SSC was used to identify live singlet cell and 10000 cells were gated using fluorescent anti-human TCR α/β antibody comparing to isotype controls.

- ☒ Tick this box to confirm that a figure exemplifying the gating strategy is provided in the Supplementary Information.
